# Cellular immune activity biomarker neopterin is associated hyperlipidemia: results from a large population-based study

**DOI:** 10.1186/s12979-016-0059-y

**Published:** 2016-02-25

**Authors:** Shu-Chun Chuang, Heiner Boeing, Stein Emil Vollset, Øivind Midttun, Per Magne Ueland, Bas Bueno-de-Mesquita, Martin Lajous, Guy Fagherazzi, Marie-Christine Boutron-Ruault, Rudolf Kaaks, Tilman Küehn, Tobias Pischon, Dagmar Drogan, Anne Tjønneland, Kim Overvad, J Ramón Quirós, Antonio Agudo, Esther Molina-Montes, Miren Dorronsoro, José María Huerta, Aurelio Barricarte, Kay-Tee Khaw, Nicholas J. Wareham, Ruth C. Travis, Antonia Trichopoulou, Pagona Lagiou, Dimitrios Trichopoulos, Giovanna Masala, Claudia Agnoli, Rosario Tumino, Amalia Mattiello, Petra H Peeters, Elisabete Weiderpass, Richard Palmqvist, Ingrid Ljuslinder, Marc Gunter, Yunxia Lu, Amanda J. Cross, Elio Riboli, Paolo Vineis, Krasimira Aleksandrova

**Affiliations:** Institute of Population Health Sciences, National Health Research Institutes, 35 Keyan Road, Zhunan, Miaoli County, 35053 Taiwan; Department of Epidemiology and Biostatistics, School of Public Health, Imperial College London, London, UK; Department of Epidemiology, German Institute of Human Nutrition Potsdam-Rehbruecke, Nuthetal, Germany; Department of Public Health and Primary Health Care, University of Bergen, Bergen, Norway; Division of Epidemiology, Norwegian Institute of Public Health, Bergen, Norway; Bevital AS, Bergen, Norway; Department of Clinical Science, University of Bergen, Bergen, Norway; Laboratory of Clinical Biochemistry, Haukeland University Hospital, Bergen, Norway; The National Institute for Public Health and the Environment (RIVM), Bilthoven, The Netherlands; Department of Gastroenterology and Hepatology, University Medical Centre, Utrecht, The Netherlands; Department of Social and Preventive Medicine, Faculty of Medicine, University of Malaya, Kuala Lumpur, Malaysia; Inserm, Centre for research in Epidemiology and Population Health (CESP), U1018, Nutrition, Hormones and Women’s Health team, F-94805 Villejuif, France; University of Paris Sud, UMRS 1018, F-94805 Villejuif, France; IGR, F-94805, Villejuif, France; Division of Cancer Epidemiology, German Cancer Research Center, Heidelberg, Germany; Molecular Epidemiology Group, Max Delbrueck Center for Molecular Medicine (MDC), Berlin-Buch, Germany; Department of Epidemiology, German Institute of Human Nutrition Potsdam-Rehbrücke, Nuthetal, Germany; Diet, Genes and Environment, Danish Cancer Society Research Center, Copenhagen, Denmark; Department of Public Health, Section for Epidemiology, Aarhus University, Aarhus, Denmark; Public Health Directorate, Asturias, Oviedo Spain; Unit of Nutrition, Environment and Cancer, Catalan Institute of Oncology-ICO, IDIBELL, L’Hospitalet de Llobregat, Barcelona, Spain; Escuela Andaluza de Salud Pública. Instituto de Investigación Biosanitaria de Granada (Granada.ibs), Granada, Spain; Consortium for Biomedical Research in Epidemiology and Public Health (CIBER Epidemiología y Salud Pública-CIBERESP), Madrid, Spain; Epidemiology and Health Information, Public Health Division of Gipuzkoa, Basque Regional Health Department, San Sebastian, Spain; Department of Epidemiology, Murcia Regional Health Council, Murcia, Spain; Navarre Public Health Institute, Pamplona, Spain; Clinical Gerontology Unit, Addenbrooke’s Hospital, University of Cambridge School of Clinical Medicine, Cambridge, UK; MRC Epidemiology Unit, Institute of Metabolic Science, University of Cambridge School of Clinical Medicine, Cambridge, UK; Cancer Epidemiology Unit, Nuffield Department of Population Health, University of Oxford, Oxford, UK; Hellenic Health Foundation, Athens, Greece; Bureau of Epidemiologic Research, Academy of Athens, Athens, Greece; Department of Hygiene, Epidemiology and Medical Statistics, University of Athens Medical School, Athens, Greece; Department of Epidemiology, Harvard School of Public Health, Boston, USA; Molecular and Nutritional Epidemiology Unit, Cancer Research and Prevention Institute – ISPO, Florence, Italy; Epidemiology and Prevention Unit, Fondazione IRCCS Istituto Nazionale dei Tumori, Milan, Italy; Cancer Registry and Histopathology Unit, “Civic - M.P. Arezzo” Hospital, ASP Ragusa, Italy; Dipartamento di Medicina Clinica e Chirurgia, Federico II University, Naples, Italy; Julius Center for Health Sciences and Primary Care, University Medical Center Utrecht, Utrecht, The Netherlands; Department of Community Medicine, Faculty of Health Sciences, University of Tromso, Tromsø, Norway; Department of Research, Cancer Registry of Norway, Oslo, Norway; Department of Medical Epidemiology and Biostatistics, Karolinska Institutet, Stockholm, Sweden; Samfundet Folkhälsan, Helsinki, Finland; Department of Medical Biosciences, Pathology, Umeå University, Umeå, Sweden; Department of Radiation Sciences, Oncology, Umeå University, Umeå, Sweden; Nutrition, Immunity and Metabolism Start-up Lab, Department of Epidemiology, German Institute of Human Nutrition Potsdam-Rehbruecke, Nuthetal, Germany

**Keywords:** Neopterin, Cell-mediated immunity, Metabolic syndrome

## Abstract

**Background:**

Increased serum neopterin had been described in older age two decades ago. Neopterin is a biomarker of systemic adaptive immune activation that could be potentially implicated in metabolic syndrome (MetS). Measurements of waist circumference, triglycerides, high-density lipoprotein cholesterol (HDLC), systolic and diastolic blood pressure, glycated hemoglobin as components of MetS definition, and plasma total neopterin concentrations were performed in 594 participants recruited in the European Prospective Investigation into Cancer and Nutrition (EPIC).

**Results:**

Higher total neopterin concentrations were associated with reduced HDLC (9.7 %, *p* < 0.01 for men and 9.2 %, *p* < 0.01 for women), whereas no association was observed with the rest of the MetS components as well as with MetS overall (per 10 nmol/L: OR = 1.42, 95 % CI = 0.85-2.39 for men and OR = 1.38, 95 % CI = 0.79-2.43).

**Conclusions:**

These data suggest that high total neopterin concentrations are cross-sectionally associated with reduced HDLC, but not with overall MetS.

## Background

Neopterin, a biomarker of systemic adaptive immune activation, is synthesized by monocyte-derived macrophages and dendritic cells upon stimulation of interferon-gamma (IFN-γ) and is considered a reliable proxy to assess the rate of IFN-γ production [1–4]. The concentrations of neopterin increase with the dose of interferon, thereby help to monitor the activity of INF-γ inducible inflammation. Thus, the measurement of neopterin concentrations in body fluids provides information about activation of T-helper cell derived systemic adaptive immune activation [5]. As high neopterin is associated with increased production of reactive oxygen species, neopterin can also be regarded as an indicator for oxidative stress due to immune activation [6].

Neopterin has been used clinically in the assessment of bacterial and viral infections, autoimmune diseases, and malignant conditions [7]. Increased blood neopterin concentrations had been described in older age [8, 9] and have been positively related to aging-related chronic disorders, including metabolic syndrome (MetS) [3], cancer, cardio-vascular disease, as well as overall mortality [2–4, 10–13].

An emerging field of research - immunometabolism - recognizes the existence of an interplay between immunity, inflammation, and impaired metabolism [14]. Central to this theory, inflammation and immune activation are involved in the development of obesity, insulin resistance and potentially also in MetS [14–16]. Despite biological plausibility, only a few epidemiological studies have explored the relation between neopterin and selected metabolic factors. In a study of 3946 patients with acute coronary syndrome, higher plasma concentrations of neopterin were associated with older age, a prior history of hypertension or diabetes, lower low-density lipoprotein cholesterol levels, and higher high-sensitivity C-reactive protein (hsCRP) levels [17]. In another study among 592 patients with high prevalence of MetS, plasma neopterin concentrations were correlated, though weakly, with abdominal obesity, high-density lipoprotein cholesterol (HDLC), and insulin resistance [2]. Similarly, a weak correlation between neopterin and abdominal obesity was reported in another patient cohort of 477 middle-aged and older white individuals at high risk for type 2 diabetes and cardiovascular disease [18].

It remains unclear whether the potential association of neopterin with MetS and its components, may be independent of age and markers of chronic inflammation such as hsCRP. Such knowledge may provide important insights into the potential link between immune activation and impaired metabolism. Therefore, the aim of the study was to investigate the association of plasma total neopterin concentrations with MetS and its components in the European Prospective Investigation into Cancer and Nutrition (EPIC) cohort.

## Results

Overall, the geometric mean of total neopterin concentrations in the study population were 18.74 (standard deviations, SD: 1.50) for men and 18.63 (SD: 1.40) for women. Table 1 shows the characteristics of the study population.

**Table 1:** Characteristics of the study population

	Total		Men		Women	
	N	%	N	%	N	%
Age, mean (SD)	57.59	(8.24)	58.24	(7.81)	57.04	(8.55)
Education						
None or primary school completed	247	42	118	44	129	40
Technical/professional school	123	21	58	21	65	20
Secondary school	83	14	16	6	67	21
Longer education	119	20	66	24	53	16
Not specified	22	4	12	4	10	3
Smoking status						
Never	273	46	80	30	193	60
Former	196	33	125	46	71	22
Current	118	20	61	23	57	18
Unknown	7	1	4	1	3	1
Physical activity						
Low	102	17	60	22	42	13
Medium	121	20	62	23	59	18
High	139	23	53	20	86	27
Very high	205	35	77	29	128	40
Missing	27	5	18	7	9	3
Waist circumference, cm, mean (SD)	87.22	(11.83)	94.37	(9.33)	81.41	(10.38)
Tryglicerides, mmol/L, mean (SD)	1.19	(0.94)	1.29	(1.07)	1.10	(0.80)
High-density lipoprotein cholesterol, mmol/L, mean (SD)	1.43	(0.39)	1.29	(0.36)	1.54	(0.38)
Systolic blood pressure, mmHg, mean (SD)	128.93	(16.77)	131.41	(15.70)	127.14	(17.31)
Diastolic blood pressure, mmHg, mean (SD)	79.72	(9.84)	81.94	(9.76)	78.12	(9.60)
HbA1c (%), mean (SD)	5.74	(0.61)	5.77	(0.63)	5.71	(0.60)
Nopterin (nmol/L) , mean (SD)	20.07	(7.90)	20.39	(8.37)	19.81	(7.49)

Table 2 presents the Spearman’s partial correlation coefficients of clinical markers of MetS and total neopterin concentrations. Total neopterin was inversely correlated with pyridoxal 5′-phosphate (PLP) and HDLC but positively correlated with hsCRP.

**Table 2:** Spearman partial correlation coefficients (r)^1^ between total neopterin, pyridoxal 5'-phosphate (PLP) and markers of metabolic factors

	PLP, μmol/L		Waist circumference, cm		Tryglicerides, mmol/L		High-density lipoprotein cholesterol, mmol/L		Systolic blood pressure (mmHg)		Diastolic blood pressure, mmHg		HbA1c (%)		hsCRP, mg/L	
	ρ	p	ρ	p	ρ	p	ρ	p	ρ	p	ρ	p	ρ	p	ρ	p
	Men															
Neopterin, nmol/L	−0.13	0.04	0.24	<0.01	−0.07	0.29	−0.18	0.01	0.09	0.22	0.16	0.04	0.01	0.91	0.18	0.01
PLP, μmol/L			0.08	0.24	−0.04	0.55	0.21	<0.01	−0.18	0.07	−0.05	0.57	−0.09	0.35	−0.11	0.11
Waist circumference, cm					0.26	<0.01	−0.23	<0.01	0.26	<0.01	0.28	<0.01	0.12	0.15	0.21	0.02
Tryglicerides, mmol/L							−0.35	<0.01	0.15	0.04	0.24	0.00	0.06	0.50	0.02	0.79
High-density lipoprotein cholesterol, mmol/L									−0.18	0.01	−0.17	0.02	0.04	0.58	−0.17	0.01
Systolic blood pressure, mmHg											0.66	<0.01	0.11	0.27	0.08	0.29
Diastolic blood pressure, mmHg													−0.01	0.89	0.07	0.36
HbA1c (%)															0.07	0.45
	Women															
Neopterin, nmol/L	−0.15	0.01	0.10	0.09	0.02	0.68	−0.24	<0.01	−0.05	0.43	−0.07	0.28	−0.11	0.23	0.15	0.01
PLP, μmol/L			−0.06	0.29	−0.08	0.17	0.14	0.02	−0.08	0.44	−0.03	0.78	0.25	0.01	−0.20	<0.01
Waist circumference, cm					0.31	<0.01	−0.28	<0.01	0.25	<0.01	0.21	<0.01	0.14	0.15	0.27	<0.01
Tryglicerides, mmol/L							−0.35	<0.01	0.16	0.01	0.13	0.03	0.07	0.50	0.15	0.12
High-density lipoprotein cholesterol, mmol/L									−0.04	0.48	0.01	0.89	0.14	0.12	−0.09	0.12
Systolic blood pressure, mmHg											0.64	<0.01	0.01	0.95	0.19	<0.01
Diastolic blood pressure, mmHg													0.03	0.76	0.04	0.56
HbA1c (%)															0.00	0.99

^1.^The partial correlation coefficients were adjusted for age at blood collection (years), sex, EPIC study centers, and smoking status (never, former, current, and unknown)

*Abbreviations: PLP* pyridoxal 5'-phosphate, *WC* waist circumference, *TG* triglycerides, *HDLC* high-density lipoprotein cholesterol, *SBP* systolic blood pressure, *DBP* diastolic blood pressure, *hsCRP* high-sensitivity C-reactive protein, *HbA1c* glycatred hemoglobin

**Fig. 1:**
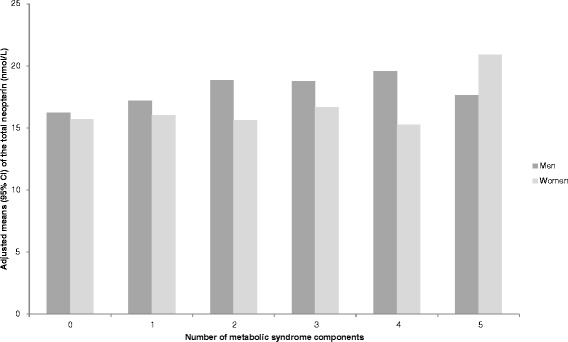
Adjusted means^1^ and 95 % confidence intervals of total neopterin by increasing number of metabolic syndrome components^2^. 1. The means were calculated by exponentiating the natural-log transformed means, which were estimated from multiple linear regression and adjusted for age at blood collection (years), sex, education (none or primary school completed, technical or professional school, secondary school, above secondary school, and not specified), smoking status (never, former, current, and unknown), and physical activity (low, medium, high, very high, missing). 2. The markers were considered abnormal when waist circumference ≥94 cm in men and ≥88 in women; triglycerides ≥1.7 mmol/L; high-density lipoprotein cholesterol <1.03 in men, <1.29 mmol/L in women; systolic blood pressure ≥130 or diastolic ≥85 mmHg; and HbA1c ≥5.7 % or self-reported ever physician diagnosed diabetes

High total neopterin concentrations were associated with lower HDLC, but not with other components of MetS (Table 3). After mutual adjustment, the mean total neopterin concentrations remained different according to HDLC categories (p < 0.01 for both men and women). Figure 1 shows the adjusted means and 95 % CI of total neopterin by increasing number of MetS components. The average differences per component was 4.0 % (*P*_difference_ = 0.07) for men and 0.8 % (*P*_difference_ = 0.64) for women.

**Table 3:** Adjusted geometric means and 95 % confidence intervals (95 % CI) of the means of total neopterin by levels of pyridoxal 5'-phosphate (PLP) and markers of metabolic factors

	Men					Women				
	Mean^1^	95 % CI		Difference^2^	*p*	Mean^1^	95 % CI		Difference^2^	*p*
PLP, μmol/L										
T1 (M: <=28.1 (Median:21.4); F: <=24.5 (Median:19.6))	18.09	15.67	20.89			15.53	13.42	17.98		
T2 (M: 28.1-44.9 (Median:34.7); F: 24.5-38.9 (Median:30.4))	17.26	14.81	20.11	−4.7 %	0.43	14.68	12.81	16.83	−7.3 %	0.11
T3 (M: >44.9 (Median:63.5); F: >38.9 (Median:53.9))	16.72	14.52	19.24	−7.9 %	0.18	16.71	14.60	19.13	−13.0 %	0.01
Per tertile				−3.9 %	0.18				−6.5 %	0.01
Waist circumference (cm)										
T1 (M: <=91.01 (Median:86.45); F: <=76.77 (Median:71.50))	17.26	14.90	19.98			16.03	13.77	18.67		
T2 (M: 91.01-98.71 (Median:95); F: 76.77-85.51 (Median:80.50))	18.93	16.37	21.89	9.2 %	0.12	16.25	13.91	18.98	1.3 %	0.77
T3 (M: >98.71 (Median:104); F: >85.51 (Median:93))	19.17	16.44	22.37	10.5 %	0.11	16.93	14.62	19.61	5.4 %	0.26
Per tertile				5.3 %	0.11				2.7 %	0.27
Triglyceride (mmol/L)										
<1.7	17.07	14.95	19.49			16.24	13.94	18.92		
1.7-3.4	17.09	14.62	19.99	0.2 %	0.98	16.21	14.01	18.75	−3.7 %	0.53
> = 3.4	21.66	16.99	27.61	23.8 %	0.03	16.29	13.94	19.03	9.3 %	0.48
Per mmol/L				5.9 %	0.03				2.3 %	0.56
High-density lipoprotein cholesterol (mmol/L)										
T1 (M: <=1.10 (median:0.98); F: <=1.33 (median:1.18))	19.72	17.09	22.75			18.52	15.97	21.47		
T2 (M: 1.10-1.34 (median:1.21); F: 1.33-1.62 (median:1.49))	16.52	14.33	19.04	−17.7 %	0.00	15.73	13.62	18.15	−16.4 %	0.00
T3 (M: >1.34 (median:1.60); F: >1.62 (median:1.87))	16.29	14.15	18.75	−19.1 %	0.00	15.44	13.36	17.85	−18.2 %	<0.01
Per tertile				−9.7 %	0.00				−9.2 %	<0.01
Low-density lipoprotein cholesterol (mmol/L)										
Q1 (M: <3.88 (median:3.37); F: <3.88 (median:3.34))	18.96	16.39	21.94			16.65	14.49	19.14		
Q2 (M: 3.88-4.70 (median:4.25); F: 3.88-4.90 (median:4.34))	16.79	14.64	19.27	−12.2 %	0.03	15.60	13.59	17.92	−6.5 %	0.17
Q3 (M: ≥4.70 (median:5.29); F: ≥4.90 (median:5.55))	16.63	14.37	19.26	−13.1 %	0.03	14.76	12.83	16.98	−12.1 %	0.02
Per tertile				−6.6 %	0.03				−6.0 %	0.02
Total cholesterol (mmol/L)										
Q1 (M: <5.74 (median:5.16); F: <5.94 (median:5.29))	19.19	16.58	22.21			16.61	14.43	19.12		
Q2 (M: 5.74-6.59 (median:6.17); F: 5.94-6.99 (median:6.49))	16.61	14.48	19.06	−14.4 %	0.01	15.47	13.46	17.79	−7.1 %	0.15
Q3 (M: ≥6.59 (median:7.32); F: ≥6.99 (median:7.69))	16.85	14.53	19.54	−13.0 %	0.03	15.03	13.07	17.29	−10.0 %	0.05
Per tertile				−6.8 %	0.03				−4.9 %	0.05
Blood pressure										
Systolic BP										
<=123	16.87	14.59	19.51			16.20	13.68	19.18		
123-139	17.01	14.66	19.74	0.8 %	0.89	16.15	13.66	19.09	−0.3 %	0.95
>139	18.14	15.50	21.24	7.3 %	0.28	15.75	13.31	18.64	−2.8 %	0.61
Per tertile				3.6 %	0.27				−1.4 %	0.60
Diastolic BP										
<=76	16.76	14.53	19.34			16.43	13.88	19.46		
76-85	16.93	14.59	19.65	1.0 %	0.87	16.11	13.68	18.98	−0.02	0.70
>85	18.50	15.85	21.60	9.9 %	0.12	15.32	12.90	18.19	−0.07	0.19
Per tertile				4.9 %	0.11				−3.5 %	0.19
Systolic BP ≥130 or diastolic BP ≥85 mmHg or diagnosis for hypertension										
No	16.89	14.60	19.54			16.01	14.06	18.25		
Yes	17.29	14.83	20.15	2.3 %	0.66	14.90	12.94	17.16	−7.2 %	0.08
HbA1C										
T1 (M < 5.5; F < 5.5)	17.96	15.72	20.50			18.24	14.72	22.60		
T2 (M: 5.5-5.9; F: 5.5-5.8)	17.00	14.81	19.51	−5.5 %	0.43	17.75	14.18	22.22	−2.7 %	0.67
T3 (M ≥ 5.9; F ≥ 5.8)	17.51	15.07	20.34	−2.5 %	0.74	16.73	13.29	21.06	−8.7 %	0.22
Per tertile				−1.4 %	0.71				−4.2 %	0.23
<5.7 %	17.77	15.61	20.24		0.67	17.85	14.44	22.08		0.84
≥5.7 %	17.32	15.30	19.62	−2.6 %		17.66	14.19	21.98	−1.1 %	
HbA1C ≥5.7 % or diagnosis for diabetes										
No	16.99	14.74	19.59		0.80	15.78	13.81	18.05		0.37
Yes	17.21	14.67	20.18	1.3 %		15.16	13.09	17.57	−4.0 %	

^1.^The means were calculated by exponentiating the natural-log transformed means, which were estimated from multiple linear regression and adjusted for age at blood collection (years), sex, country, education (none or primary school completed, technical or professional school, secondary school, above secondary school, and not specified), smoking status (never, former, current, and unknown), and physical activity (low, medium, high, very high, missing)

^2.^The differences compared to the first category of each variable

*Abbreviations: T* tertile, *PLP* pyridoxal 5'-phosphate, *BP* blood pressure, *HbA1C* glycated hemoglobin

In our study, increased total neopterin was associated with reduced HDLC (OR _per 10 nmol/L_ = 2.22, 95 % CI = 1.24-3.97 for men and OR _per 10 nmol/L_ = 2.82, 95 % CI = 1.68-4.73 for women), and these associations were independent of PLP (Table 4). Further adjustment for hsCRP did not change the results (OR _per 10 nmol/L_ = 2.14, 95 % CI = 1.17-3.91 for men and OR _per 10 nmol/L_ = 2.70, 95 % CI = 1.58-4.61, data not shown). Increased plasma total neopterin was not associated with overall MetS, defined as presence of any three of the MetS components (OR _per 10 nmol/L_ = 1.42, 95 % CI = 0.85-2.39 for men and OR _per 10 nmol/L_ = 1.38, 95 % CI = 0.79-2.43 for women).

**Table 4:** Association between total neopterin and metabolic syndrome (MetS)^1^ and its components

MetS components	Total Neopterin, nmol/L, Men																
	T1^2^			T2^2^					T3^2^					Per 10 nmol/L			
	Normal	Abnormal	OR^4^	Normal	Abnormal	OR^4^	95 % CI		Normal	Abnormal	OR^4^	95 % CI		OR^4^	95 % CI		*P*_*trend*_
Waist circumference ≥94 cm in men	52	37	1.00	39	51	2.00	(1.00,	3.99)	31	49	2.12	(1.01,	4.42)	1.64	(0.99,	2.73)	0.05
Triglycerides ≥1.7 mmol/L	77	22	1.00	67	22	1.21	(0.57,	2.56)	58	22	1.14	(0.52,	2.50)	1.08	(0.64,	1.84)	0.77
High-density lipoprotein cholesterol ,<1.03 in men	90	9	1.00	69	21	2.75	(1.13,	6.70)	56	24	3.71	(1.48,	9.32)	2.22	(1.24,	3.97)	0.01
Systolic blood pressure ≥130 or diastolic ≥85 mmHg or diagnosis for hypertension	49	50	1.00	50	40	0.93	(0.43,	2.04)	37	43	0.82	(0.34,	1.95)	0.87	(0.48,	1.59)	0.65
HbA1c ≥5.7 % or self-reported ever physician diagnosed diabetes	69	30	1.00	53	37	1.62	(0.83,	3.15)	50	30	1.47	(0.72,	3.01)	1.28	(0.79,	2.08)	0.32
Any three of the above	86	13	1.00	73	18	2.02	(0.95,	4.30)	61	19	1.82	(0.83,	3.99)	1.42	(0.85,	2.39)	0.18
MetS components	Total Neopterin, nmol/L, Women																
	T1^2^			T2^2^					T3^2^					Per 10 nmol/L			
	Normal	Abnormal	OR^4^	Normal	Abnormal	OR^4^	95 % CI		Normal	Abnormal	OR^4^	95 % CI		OR^4^	95 % CI		*P*_*trend*_
Waist circumference ≥80 cm in women^3^	68	51	1.00	49	57	1.48	(0.83,	2.61)	44	50	1.27	(0.70,	2.31)	1.21	(0.76,	1.94)	0.43
Triglycerides ≥1.7 mmol/L	104	17	1.00	90	15	0.97	(0.43,	2.16)	80	15	0.87	(0.38,	2.00)	0.89	(0.46,	1.73)	0.74
High-density lipoprotein cholesterol, <1.29 mmol/L in women	102	19	1.00	72	34	2.90	(1.48,	5.70)	58	37	4.05	(2.04,	8.03)	2.82	(1.68,	4.73)	<0.01
Systolic blood pressure ≥130 or diastolic ≥85 mmHg or diagnosis for hypertension	73	50	1.00	64	42	0.80	(0.43,	1.51)	60	35	0.66	(0.33,	1.30)	0.72	(0.42,	1.23)	0.22
HbA1c ≥5.7 % or self-reported ever physician diagnosed diabetes	90	30	1.00	80	24	0.90	(0.46,	1.74)	70	25	1.08	(0.55,	2.10)	1.06	(0.62,	1.81)	0.83
Any three of the above	109	14	1.00	90	16	1.43	(0.71,	2.91)	77	18	1.53	(0.74,	3.16)	1.38	(0.79,	2.43)	0.26

^1.^Metabolic syndrome is defined based on the joint interim statement of the International Diabetes Federation Task Force on Epidemiology and Prevention; National Heart, Lung, and Blood Institute; American Heart Association; World Heart Federation; International Atherosclerosis Society; and International Association for the Study of Obesity. Analysis is based on an alternate MetS definition modified to include HbA1C instead of glucose as a marker for impaired glucose metabolism (22)

^2.^Tertile 1 (T1, nmol/L): ≤17.20 for men and ≤16.30 for women; tertile 2 (T2): 17.20-22.60 for men and 16.30-21.90 for women; tertile 3 (T3): >22.60 for men and >21.90 for women. Medians (nmol/L): 13.70 for men and 14.18 for women in T1, 19.60 for men and 19.65 for women in T2, and 28.20 for men and 26.80 for women in T3

^3.^Recent American Heart Association/National Heart, Lung, and Blood Institute guidelines for metabolic syndrome recognize an increased risk for CVD and diabetes at waist-circumference thresholds of ≥94 cm in men and ≥80 cm in women and identify these as optional cut points for individuals or populations with increased insulin resistance (22)

^4.^ORs were adjusted for age at blood collection (years), sex, country, education (none or primary school completed, technical or professional school, secondary school, above secondary school, and not specified), smoking status (never, former, current, and unknown), and physical activity (low, medium, high, very high, missing)

*Abbreviations: MetS* metabolic syndrome, *T* tertile, *OR* odds ratio

## Discussion

In this study, high total neopterin concentrations were associated with reduced HDLC, but not with overall MetS. These data indicate that immune activation may be related to lipid changes; however, the cross-sectional nature of the study does not provide sufficient information for interpreting the direction of these relations.

Previously only three studies investigated the association of neopterin concentrations with clinical markers of MetS; however, these studies were conducted in participants with underlying diseases, including cardiovascular disease, type 2 diabetes and MetS [2, 18, 19]. In agreement with the study of Oxengrug et al. 2011 [2], we observed an inverse association of total neopterin with HDLC.

Our data suggest an inverse association of total neopterin with HDLC, as well as low-density lipoprotein cholesterol and total cholesterol. This association was independent of age, sex, EPIC study center and smoking status as well as PLP and hsCRP levels. Similar findings have been reported, in patients with HIV infection [20], cardiovascular diseases [4] and MetS [2]. HDLC helps to remove excess cholesterol from peripheral tissue and transport it to the liver for excretion. The functions of HDL include anti-inflammatory and anti-oxidant activities [21]. If the function is impaired, cholesterol accumulates in peripheral tissue and causes inflammation and atherosclerosis. Despite the concept of HDL dysfunction evolved over the last decades, little is known on factors that underline possible alterations between functional and dysfunctional HDL. Recently, immunity was suggested as one of the main pathophysiological pathways of HDLC functionality via modulating cholesterol content in immune cells [22]. It has been shown that inhibition of cholesterol efflux mechanisms in macrophages promotes an inflammatory phenotype in these cells [23]. The raised neopterin levels may indicate activated immune response in individuals with low HDL cholesterol levels. Neopterin had been associated with cardiovascular events [4, 24, 25], suggesting a potential involvement of adaptive immunity and inflammation in modulating the association between cholesterol metabolism and cardio-metabolic risk. From a practical perspective, measurement of neopterin in addition to HDLC may aid in identifying HDL anti-inflammatory/proinflammatory function and could likely yield important additional information beyond that available from simple measurement of HDLC in an individual. However, future studies are needed in order to evaluate potential practical implication of these findings. Of note, despite the association with HDLC, we observed no association with previous diagnosis of hyperlipidemia (data not shown). This can be partly explained by the observation that total neopterin concentrations were lower in those who used lipid-lowering drugs [4].

Previous studies have reported positive associations between neopterin and waist circumference [2, 10, 18, 26, 27]. In one of these; however, such association disappeared after adjustment for other metabolic biomarkers [18]. Thewissen et al. (2011) reported that the association between abdominal fat and neopterin - considered a marker of adaptive immune activation - was mediated, by elevations in hsCRP and other immune activation markers [18]. They hypothesized that it is not merely an increased mass of adipose tissue that directly leads to attenuation of insulin action, but rather adipose tissue inflammation mediated by activated immune system in obese individuals that leads to insulin resistance. In our study, we only observed a non-statistically significant marginal association between abdominal obesity and total neopterin concentrations. Further prospective studies are needed to test this hypothesis.

Similarly to a previous report [4], we found no association between total neopterin concentrations and triglycerides (TG). There was no association between total neopterin concentrations and measured systolic or diastolic blood pressure (BP), including pre-defined cutoffs for hypertension. There have been reports suggesting that neopterin could be a predictive marker for cardiovascular events [4, 19, 24, 25, 28–31], including an elevated diastolic BP [29, 32]. However, its associations with hypertension (or BP) have been inconsistent across studies. In this context, our results might suggest that although hypertension is an important component of cardiovascular diseases, it might not be directly associated with inflammation or IFN-γ mediated inflammation.

Previous studies have also reported that neopterin concentrations were positively associated with glucose concentrations [10]. However, we did not observe an association between total neopterin concentrations and diabetes, either using the glycated haemoglobin (HbA1C), a marker of long-term blood glucose levels, or with self-reported diabetes. Similar findings have been obtained in a small saline-controlled crossover study on six healthy men (mean age 22 years) for IFN-γ [33].

Limitations of the present study have to be taken into account. First, the mean and median concentrations of total neopterin in this study population was somewhat higher than previously reported [2, 27]. An explanation is that our assay measures total neopterin, which is the sum of 7,8-dihydroneopterin and neopterin, in contrast to ELISA method which measures only neopterin. Nevertheless, both neopterin and total neopterin reflect inflammation and the associations between total neopterin and hsCRP, as well as the other metabolic biomarkers were comparable with previous reports (9). In addition, in our data no unexpected correlations between neopterin and basic characteristics were observed, as well as main findings were also in line with the previous reports. Secondly, the study population included controls of a nested case–control study; therefore, it may not be representative of the general population. However, when compared to the overall EPIC population, we have not seen major differences according to baseline characteristics, except for that our study population was slightly older, included a higher proportion of men, and a higher proportion of smokers. The range of the concentration of total neopterin reported here may not be fully representative of the general population. Thirdly, the relation between total neopterin and MetS components was assessed within the context of a cross-sectional study design, which does not allow inference about the direction of the associations. Finally, about 70 % of the study participants provided non-fasting blood samples, which may have affected the TG levels; however, we have been accounting for fasting status and found essentially the same results after excluding non-fasting participants.

## Conclusions

In conclusion, high total neopterin concentrations are associated with reduced HDLC, but not with overall MetS. These data support the emerging knowledge on the interplay of immune response and cholesterol metabolism. Future studies are warranted to better understand the potential role of these interrelations in chronic disease development.

## Methods

### Study population

The design of the EPIC cohort has been described previously [34]. In brief, EPIC recruited 518,408 volunteers from 23 centers in 10 countries (Sweden, Denmark, Norway, the Netherlands, United Kingdom, France, Germany, Spain, Italy, and Greece) between 1992 and 2000. The eligibility criteria for participation was primarily decided within each cohort. In general, apparently healthy, middle-aged subjects who agreed to participate in the study and to have their health status followed up for the rest of their lives, were recruited. The questionnaires were completed and the blood samples were taken at recruitment.

### Assessment of anthropometry and lifestyle data

The lifestyle questionnaires, which were completed by participants, included questions on diet, education, occupation, previous illnesses, alcohol, tobacco consumption, and physical activity. Informed consent forms were filled at each local center and the study was approved by the Institutional Review Board at the International Agency for Research on Cancer (IARC) and the local ethics committees. Waist circumference was measured either at the narrowest torso circumference or at the midpoint between the lower ribs and iliac crest. Systolic BP and diastolic BP were measured by trained personnel. Two readings were performed on the right arm in a sitting position (spaced by 1–5 minutes) by use of a standard mercury manometer or oscillometric device. To avoid any possible white coat effect, we used the second reading, and when unavailable, the first reading.

### Definition of MetS

The definition of MetS and its components have been described previously [35]. In general, we followed the harmonized definition published by Alberti et al. in 2009 [36] with slight modification in determining abnormal glucose metabolism. Briefly, MetS was defined as having any three of the following five components: 1) abdominal obesity, i.e. waist circumference is greater than or equal to 94 cm in men or 80 cm in women; 2) elevated TG, i.e. greater than or equal to 1.7 mmol/L, after correction for the fasting status of the study subjects; that is, subtracting the sex-specific geometric mean difference between non-fasting and fasting subjects from the individual levels of non-fasting subjects; 3) reduced HDLC, i.e. less than 1.03 mmol/L in men and 1.29 mmol/L in women; 4) elevated BP, i.e. systolic BP 130 mmHg or more or diastolic BP 85 mmHg or more or self-reported physician diagnosed hypertension; and 5) abnormal glucose metabolism, i.e. self-reported physician diagnosed diabetes status or HbA1c of 5.7 %, which corresponds to fasting plasma glucose levels of 100 mg/dL .

### Laboratory assays

Plasma concentration of total neopterin (7,8-dihydroneopterin + neopterin) and PLP was determined by liquid chromatography-tandem mass spectrometry (LC-MS/MS) [37] at Bevital A/S (http://www.bevital.no), Bergen, Norway. Serum hsCRP was measured by a high-sensitivity assay (Beckman-Coulter, Woerden, the Netherlands), and the HDLC and TG concentrations by a colorimetric method, on a Synchron LX-20 Pro autoanalyzer (Beckman-Coulter, [38]). Measurements of HbA1c in erythrocyte hemolysate were carried out using high-performance liquid chromatography with a Bio-Rad Variant II instrument (Bio-Rad Laboratories, Hercules, California) [39]. The within and between day coefficients of variance (CV) were 3-10 % for total neopterin and PLP [37]. The inter-assay CV were 6.0 % and 6.5 % at CRP concentrations of 1.16 and 1.89 mg/L, respectively, 4.1 %, 3.4 %, and 3.6 % at HDLC concentrations of 0.62, 1.20, and 1.65 mmol/L, respectively, and 3.3 %, 2.1 %, and 2.0 % at TG concentrations at 86.6, 165.9, and 227.0 mg/dL, respectively. The intra-batch CV was 2.5 % for HbA1c [39].

### Statistical analysis

The current analysis is based on 845 subjects (375 men and 470 women) who served as controls in matched case–control studies of colorectal cancer nested within the EPIC. The original aims of the nested case–control studies were to explore the risk of colon and rectal cancer in relation to MetS [35] and one-carbon metabolism biomarkers [40, 41]. MetS component measurements were not available subjects from Norway and Malmo center of Sweden. We further excluded 207 subjects who received treatment or the treatment information were missing for hyperlipidemia (*n* = 94), hypertension (*n* = 175), or diabetes (*n* = 15). Additional 44 subjects were excluded because their total neopterin measurements were not available. The final sample size for the analysis was 594.

The correlation between total neopterin and components were examined by Spearman’s partial correlation coefficients (r), adjusted for age, sex, country, and smoking status. Adjusted means for total neopterin according to tertiles of each MetS component were calculated using multiple linear regression models. Because the range of the middle category of TG is narrow, we categorized TG at 1.7 and 3.4 mmol/L. The dependent variable, total neopterin concentrations, was natural log-transformed and the normality assumption was tested by graphic examination of the residual distribution. The models were adjusted for age at blood collection (years), sex, country, education (none or primary school completed, technical or professional school, secondary school, above secondary school, and not specified), smoking status (never, former, current, and unknown), and physical activity (low, medium, high, very high, and missing). The adjusted means were also assessed by mutual adjustment for the other MetS components as well as PLP, due to its role in INF-γ stimulated inflammatory responses [2, 27] and hsCRP, due to its association with low-grade inflammation. The adjusted means were then back-transformed by exponentiating the natural-log transformed means from the model. The associations between total neopterin and pre-defined cutoffs of each component of MetS and the composite MetS were also examined by calculating odds ratios (OR) and 95 % confidence intervals (CI) in logistic regression analysis, adjusted for age at blood collection (years), sex, country, education (none or primary school completed, technical or professional school, secondary school, above secondary school, and not specified), smoking status (never, former, current, and unknown), and physical activity (low, medium, high, very high, missing). Total neopterin was modeled in three categories according to sex-specific tertiles. Tests for trend were performed by modelling the median values of each category as a continuous variable. Subgroup analyses were performed by age (<55, 50–65, and ≥65 years old), sex, and body mass index (BMI, <30 and ≥30 kg/m^2^).

Analyses were performed using SAS 9.3. All tests were two sided and statistical significance was assessed at the level of 0.05.

## Abbreviation

AGM: BP: blood pressure
CI: confidence interval
CV: coefficients of variance
EPIC: European Prospective Investigation into Cancer and Nutrition
HbA1c: glycated haemoglobin
HDLC: high-density lipoprotein cholesterol
hsCRP: high-sensitivity C-reactive protein
IFN-γ: Interferon-gamma
MetS: metabolic syndrome
OR: odds ratio
PLP: pyridoxal 5′-phosphate
TG: Triglycerides
